# Epidemiology and Mortality of Liver Abscess in End-Stage Renal Disease Dialysis Patients: Taiwan National Cohort Study

**DOI:** 10.1371/journal.pone.0088078

**Published:** 2014-02-14

**Authors:** Chon-Seng Hong, Kun-Ming Chung, Po-Chang Huang, Jhi-Joung Wang, Chun-Ming Yang, Chin-Chen Chu, Chung-Ching Chio, Fu-Lin Chang, Chih-Chiang Chien

**Affiliations:** 1 Department of Internal Medicine, Chi-Mei Medical Center, Tainan, Taiwan; 2 Department of General Medicine, Chi-Mei Medical Center, Tainan, Taiwan; 3 Department of Orthopedics Medicine, Chi-Mei Medical Center, Taiwan; 4 Department of Medical Research, Chi-Mei Medical Center, Tainan, Taiwan; 5 Department of Neurology, Chi-Mei Medical Center, Tainan, Taiwan; 6 Department of Neurological Surgery, Chi-Mei Medical Center, Tainan, Taiwan; 7 Department of Pharmaceutical Science and Technology, Chung Hwa University of Medical Technology, Tainan, Taiwan; 8 Department of Nephrology, Chi-Mei Medical Center, Tainan, Taiwan; 9 Department of Food Nutrition, Chung Hwa University of Medical Technology, Tainan, Taiwan; National Taiwan University Hospital, Taiwan

## Abstract

**Background and Objectives:**

To determine the incidence rates and mortality of liver abscess in ESRD patients on dialysis.

**Design, Setting, Participants, & Measurements:**

Using Taiwan’s National Health Insurance Research Database, we collected data from all ESRD patients who initiated dialysis between 2000 and 2006. Patients were followed until death, end of dialysis, or December 31, 2008. Predictors of liver abscess and mortality were identified using Cox models.

**Results:**

Of the 53,249 incident dialysis patients identified, 447 were diagnosed as having liver abscesses during the follow-up period (224/100,000 person-years). The cumulative incidence rate of liver abscess was 0.3%, 1.1%, and 1.5% at 1 year, 5 years, and 7 years, respectively. Elderly patients and patients on peritoneal dialysis had higher incidence rates. The baseline comorbidities of diabetes mellitus, polycystic kidney disease, malignancy, chronic liver disease, biliary tract disease, or alcoholism predicted development of liver abscess. Overall in-hospital mortality was 10.1%.

**Conclusions:**

The incidence of liver abscess is high among ESRD dialysis patients. In addition to the well known risk factors of liver abscess, two other important risk factors, peritoneal dialysis and polycystic kidney disease, were found to predict liver abscess in ESRD dialysis patients.

## Introduction

Liver abscess is associated with significant morbidity, mortality, and increased consumption of healthcare resources [1]–[3]. Several studies [1]–[4] report the incidence of liver abscess to be 1.1–3.3 per 100,000 person-years in general Western populations. Liver abscess, which is highly endemic in Taiwan, has an incidence of 11.5–17.5 per 100,000 person-years [5]. Additionally, renal failure is a significant risk factor for liver abscess [5].

The precise incidence of liver abscess has not been investigated in end-stage renal disease (ESRD) dialysis patients. ESRD patients are known to be at increased risk of infectious diseases [6], [7], and associated with high mortality [8], [9]. Liver abscess is the most common extra-renal abscess among this population [10]. One study from a general population [5] reported that patients with renal disease had three times the risk of liver abscess compared to general population. Yang et al. [11] reported in a hospital-based study using retrospective chart review, that 27 of 20,676 admitted patients with ESRD had liver abscess. However, no any longitudinal cohort study have investigated the precise incidence of liver abscess in ESRD dialysis patients.

Although the global prevalence and incidence of ESRD dialysis patients has recently increased markedly [12], [13], there are no published epidemiological data on subject of increased risked of liver abscess using a national cohort of dialysis patients. Nearly half of ESRD patients have DM. In addition, they are characteristically older in age and at increased risk of infectious diseases. We hypothesized that this patient population would have a high incidence of liver abscess. To find out, we used a national cohort longitudinal follow-up design to calculate the incidence of this disease in ESRD patients receiving dialysis. We trapped a large data set, Taiwan’s National Health Insurance Research Database (NHIRD), to perform a nationwide investigation of the epidemiology, incidence, and mortality of liver abscess in ESRD dialysis patients.

## Methods

### Database

For this study, we retrieved ambulatory care claims, all inpatient claims, and the updated registry for all ESRD patients receiving dialysis from 1998 to 2008. Data was collected from Taiwan’s NHIRD provided by Taiwan’s National Health Insurance (NHI), a compulsory universal health insurance program which has covered the healthcare costs of all of Taiwan’s residents except of prison inmates since 1995. The program requires all medical institutions to use standard computerized claim documents for reimbursement of medical expenses. Patients with end-stage renal disease (ESRD) are eligible for every type of renal replacement therapy without any charge, and all their expenses are covered by NHI. The NHIRD contains nearly all (99%) inpatient and outpatient medical benefit claims for the 23 million residents of Taiwan, and has been used extensively in various studies [14]–[17]. This database provides a great deal of information, including gender, birth date, dates of admission and discharge, the medical institutions providing the services, the ICD-9-CM (International Classification of Diseases, 9th Revision, Clinical Modification) diagnostic and procedure codes (up to five each), and encrypted outcomes. NHIRD is released with de-identified secondary data for public research purposes. All personal identification information on files connected with the present study was scrambled using surrogate identification numbers to ensure patient confidentiality. The Bureau of National Health Insurance approves the application (NHRI-NHIRD-99182), and the institutional review board of Chi-Mei Medical Center waived the need for approval.

### Patient Selection and Definition

This longitudinal cohort study selected adult ESRD patients (≥18 years old) on maintenance dialysis who began renal replacement therapy between January 1, 2000, and December 31, 2006. ESRD patients on maintenance dialysis were defined as having undergone dialysis for more than 90 days. Patients who had undergone renal transplantation before beginning dialysis were excluded. Patients were followed-up from the first reported date of dialysis to the date of death, end of dialysis, or December 31, 2008. The data of 53,249 incident dialysis patients were analyzed.

We linked study subjects to their inpatient claim data to identify the first episode of liver abscess. Cases were selected by using the following criteria: the 3 primary discharge diagnoses included liver abscess (ICD-9-CM 572.0). A total of 447 patients were diagnosed with liver abscess during the follow-up period.

### Demographic and Comorbid Variables

We linked to the diagnostic codes through the inpatient and outpatient claims databases of the NHIRD. We collected survival status, date of death, patient demographics, and baseline comorbidities. Baseline comorbidities, which were diabetes mellitus (DM), polycystic kidney disease (PCKD), malignancy, chronic liver disease (CLD), biliary tract disease, and alcoholism, are important factors affecting episodes of liver abscess [1]–[5], [11] and were assessed at the start of dialysis.

### Statistical Analysis

The incidence of newly diagnosed liver abscess was expressed as the number of cases of liver abscess per 100,000 person-years. Parametric Pearson’s chi square test was used to compare each variable in the groups of patients with and without liver abscess. Age was entered as a categorical variable (18–44, 45–64, and 65 years or older). Significance was set at p<0.05. The cumulative proportion of patients with liver abscess and of survivors after liver abscess were calculated using the Kaplan-Meier method. The log rank test was used to analyze significance. Cox proportional hazards models were used to identify the risk factors of liver abscess and mortality after liver abscess. Hazard ratios (HRs) and 95% confidence intervals (CIs) were derived from Cox proportional hazards models. Cox models met the assumption of proportionality of risks. To adjust for potential confounding in the relationship between comorbidities and the risk of mortality, multivariate analyses were used to model to all-cause mortality. The Statistical Package for Social Sciences for Windows 17.0 (SPSS Inc; Chicago, IL, USA) was used for all statistical analyses.

## Results

### Demographics and Clinical Characteristics

A total 53249 adult incident dialysis patients were enrolled in this study. Of these patients, 447 patients (224/100,000 person-years) had liver abscess during the follow-up period (Table 1). Elderly patients had higher incidence of liver abscesses. Only 0.5% of the patients 18–44 years old and 0.9% of those ≥65 years old had liver abscess (p = 0.003). Patients with liver abscess tended to have more comorbidities than those without it. Many more patients with liver abscess than without it had DM, PCKD, malignancy, CLD, biliary tract disease, and alcoholism. Mean in-hospital stays was 22.74±17.56 days. During the study period, there were 211 and 18,612 deaths in patients with and without liver abscess group, respectively.

**Table 1:** Patient characteristics and association with (n = 447) and without (n = 52802) liver abscess among end-stage renal disease dialysis patients.

	Without LA	(n = 52,802)	With LA	(n = 447)	P
	n	(%)	n	(%)	
Sex					0.283
Female	27242	(99.1)	242	(0.9)	
Male	25560	(99.2)	205	(0.8)	
Age (years)					0.003
18–44	7421	(99.5)	39	(0.5)	
45–64	23125	(99.2)	197	(0.8)	
≥65	22256	(99.1)	211	(0.9)	
Dialysis modality					0.55
PD	3749	(99.1)	35	(0.9)	
HD	49053	(99.2)	412	(0.8)	
Diabetic mellitus					<0.001
No	26896	(99.3)	190	(0.7)	
Yes	25906	(99)	257	(1)	
Coronary artery disease					0.864
No	40462	(99.2)	341	(0.8)	
Yes	12340	(99.1)	106	(0.9)	
Cerebrovascular disease					0.027
No	46245	(99.2)	376	(0.8)	
Yes	6557	(98.9)	71	(1.1)	
Chronic obstructive lung disease					0.299
No	47779	(99.2)	398	(0.8)	
Yes	5023	(99)	49	(1)	
Malignancy					0.03
No	49520	(99.2)	404	(0.8)	
Yes	3282	(98.7)	43	(1.3)	
Polycystic kidney disease					0.084
No	52026	(99.2)	436	(0.8)	
Yes	776	(98.6)	11	(1.4)	
Chronic liver disease					<0.001
No	41935	(99.3)	306	(0.7)	
Yes	10867	(98.7)	141	(1.3)	
Biliary tract disease					<0.001
No	52525	(99.2)	435	(0.8)	
Yes	277	(95.8)	12	(4.2)	
Alcoholism					0.001
No	52484	(99.2)	439	(0.8)	
Yes	318	(97.5)	8	(2.5)	
Death	18612	(35.4)	211	(47.3)	<0.001

Data are n (%) unless otherwise indicated. LA: liver abscess. PD: Peritoneal dialysis; HD: Hemodialysis.

### Cumulative Incidence and Risk Factors for Liver Abscess

**Figure 1:**
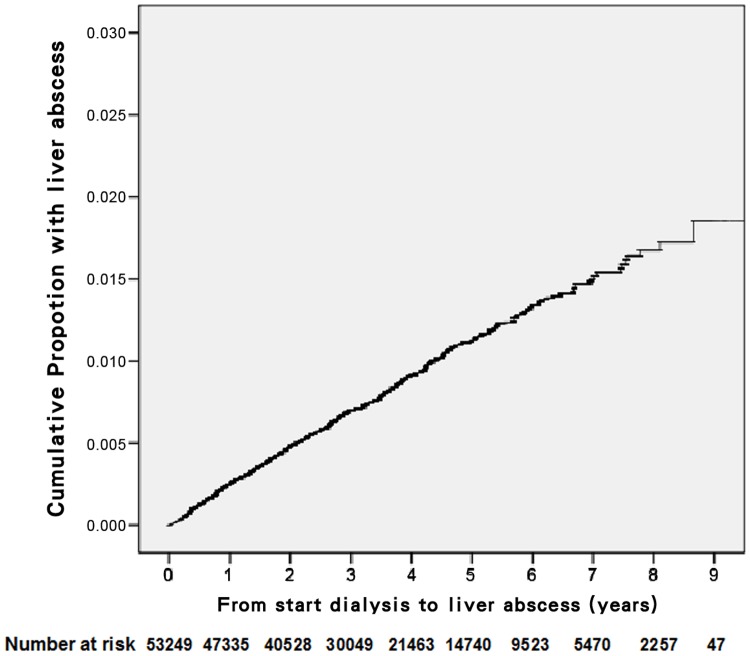
Cumulative proportion of liver abscess in end-stage renal disease dialysis patients.

The cumulative incidence rates of liver abscess in ESRD dialysis patients were 0.3% at one year, 0.7% at three years, 1.1% at five years, and 1.5% at seven years (Figure 1). After multivariate adjustment, no significant difference was found between male and female patients (Table 2). Patients ≥65 years had more than twice the incidence of liver abscess than those 18–44 years old (HR 2.33, 95% CI: 1.64–3.32). Patients on hemodialysis (HD) had a lower rate of liver abscess than those on peritoneal dialysis (PD) (HR 0.66, 95% CI: 0.46–0.93). Additionally, the following predicted a higher incidence of liver abscess: DM (HR 1.72, 95% CI: 1.41–2.09), PCKD (HR 1.99, 95% CI: 1.09–3.64), malignancy (HR 1.81, 95% CI: 1.32–2.49), CLD (HR 1.76, 95% CI: 1.44–2.17), biliary tract disease (HR 4.21, 95% CI: 2.36–7.51) or alcoholism (HR 2.617, 95% CI: 1.28–5.37).

**Table 2:** Risk factor for liver abscess after initiation of dialysis in end-stage renal disease dialysis patients (n = 53,249).

Factors	Univariate	Multivariate
	HR (95% CI)	HR (95% CI)
Sex (Male vs. Female)	0.954 (0.792–1.149)	0.922 (0.764–1.112)
Age (years)		
<45 (Referent)	1	1
45–64	1.826 (1.295–2.575)*	1.577 (1.110–2.240)*
≥65	2.597 (1.842–3.662)*	2.333 (1.638–3.323)*
Dialysis modality (HD vs. PD)	0.899 (0.637–1.270)	0.656 (0.461–0.932)*
Comorbidities		
Diabetic mellitus (yes vs. no)	1.711 (1.416–2.066)*	1.718 (1.414–2.087)*
PCKD (yes vs. no)	1.620 (0.890–2.947)	1.987 (1.086–3.636)*
Malignancy (yes vs. no)	1.978 (1.444–2.709)*	1.809 (1.316–2.486)*
Chronic liver disease (yes vs. no)	1.784 (1.461–2.178)*	1.764 (1.436–2.165)*
Biliary tract disease (yes vs. no)	5.680 (3.201–10.08)*	4.211 (2.362–7.508)*
Alcoholism (yes vs. no)	3.827 (1.901–7.701)*	2.617 (1.277–5.365)*

*HR adjusted for sex, age, dialysis modality, diabetic mellitus, polycystic kidney.

disease, malignancy, chronic liver disease, biliary tract disease and alcoholism.

*HR: Hazard ratio; CI: Confidence interval; PD: Peritoneal dialysis; HD: Hemodialysis; PCKD: polycystic kidney disease.

### Cumulative Survival Rate and Risk Factors for All-cause-mortality

**Figure 2:**
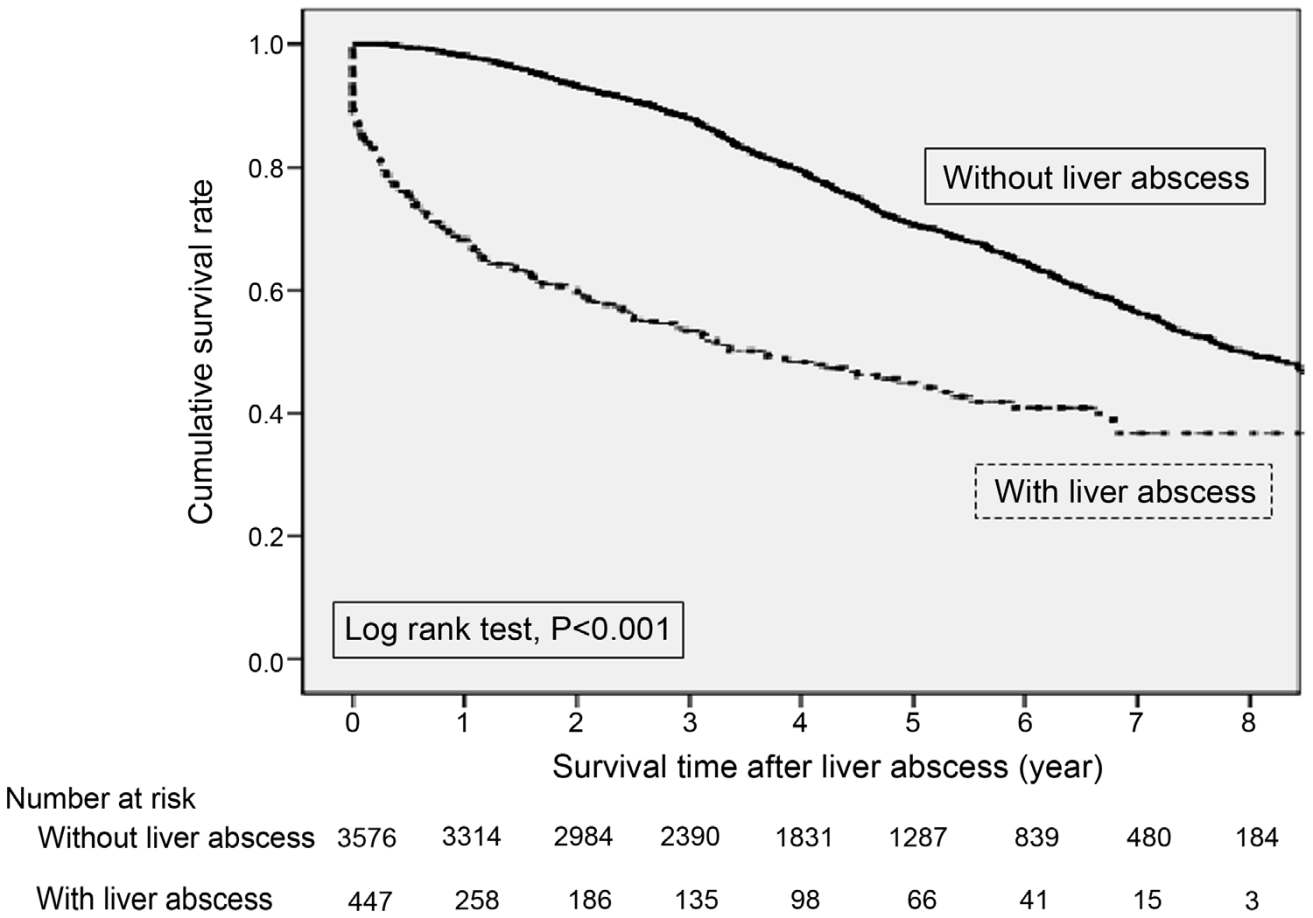
Overall survival curves after liver abscess in end-stage renal disease dialysis patients stratified by with and without liver abscess. For the control group (patients without liver abscess), patients’ age, sex, mode of dialysis and comorbidities were matched to the liver abscess group.

Overall in-hospital mortality of liver abscess was 10.14% (Figure 2). Survival curves after liver abscess were stratified by those with and without liver abscess. For the control group (patients without liver abscess), patients’ age, sex, mode of dialysis and comorbidities were matched to the liver abscess group. Patients with DM had a 64% higher risk of long-term death after liver abscess (HR 1.64, 95% CI: 1.19–2.25) than those without DM (Table 3). Additionally, having CLD was also associated with significantly higher long-term mortality after liver abscess (HR 1.62, 95% CI: 1.17–2.25).

**Table 3:** Risk factor for long-term mortality after liver abscess in end-stage renal disease dialysis patients (n = 447).

Factors	Univariate analysis	Multivariate analysis
	HR (95% CI)	HR (95% CI)
Sex (Male vs. Female)	1.083 (0.825–1.422)	1.082 (0.820–1.427)
Age (years)		
<45 (referent)	1	1
45–64	1.111 (0.552–2.236)	0.965 (0.465–2.000)
≥65	2.125 (1.082–4.172)*	1.975 (0.971–4.008)
Dialysis modality (HD vs. PD)	1.721 (0.912–3.249)	1.461 (0.764–2.795)
Comorbidities		
Diabetic Mellitus (yes vs. no)	1.545 (1.156–2.065)*	1.639 (1.193–2.252)*
Coronary artery disease (yes vs. no)	1.325 (0.981–1.789)	1.166 (0.840–1.618)
Cerebrovascular disease (yes vs. no)	1.354 (0.959–1.913)	1.149 (0.804–1.642)
COPD (yes vs. no)	0.877 (0.558–1.377)	0.832 (0.526–1.316)
Malignancy (yes vs. no)	1.377 (0.906–2.092)	1.093 (0.702–1.704)
PCKD (yes vs. no)	0.345 (0.086–1.389)	0.533 (0.129–2.195)
Chronic liver disease (yes vs. no)	0.929 (0.112–7.677)	1.622 (1.170–2.249)*
Biliary tract disease (yes vs. no)	2.016 (0.992–4.094)	1.729 (0.836–3.587)
Alcoholism (yes vs. no)	1.540 (0.570–4.157)	1.834 (0.632–5.322)

*HR adjusted for sex, age, dialysis modalities, diabetic mellitus, congestive heart failure, coronary artery disease, cerebrovascular accident, chronic obstructive pulmonary disease, polycystic kidney disease, malignancy, chronic liver disease, biliary tract disease and alcoholism.

*HR: Hazard ratio; CI: Confidence interval; PD: Peritoneal dialysis; HD: Hemodialysis; COPD: chronic obstructive pulmonary disease; PCKD: polycystic kidney disease.

*P<0.05.

## Discussion

This study represents the first nationwide population-based epidemiological study to investigate the incidence of liver abscess in chronic dialysis patients. ESRD patients receiving dialysis had a significantly higher incidence of liver abscess than general population. In addition to the well-known risk factors of liver abscess, this study was the first to report both peritoneal dialysis and polycystic kidney disease to be important risk factors for liver abscess in ESRD dialysis patients.

ESRD patients receiving dialysis have a significantly higher incidence of liver abscess. Previous studies [1]–[4] report an annual incidence of liver abscess to be about 1.1–3.3 per 100,000 person-years in general populations in Denmark, Canada, and the United States (Table 4). In Taiwan, Tsai et al. [5] reported that incidence to be 17.59 per 100,000 person-years. *Klebsiella pneumoniae* liver abscess is an endemic health problem in the East Asia and is emerging as a global disease [18]. In Taiwan, *Klebsiella pneumoniae* is the primary pathogen and DM is a major predisposing factor of liver abscess [5]. Renal disease is also a strong risk factor of liver abscess. Neutrophil dysfunction and the risk of infection are higher in ESRD dialysis patients than in the general population [6], [7]. This study found that ESRD patients receiving dialysis to have much higher incidence of liver abscess, around 224 per 100,000 person-years. Diabetic nephropathy is the leading cause of ESRD, accounting for 40–50% of patients on maintenance dialysis [16]. Therefore, it is no surprise that the incidence of liver abscess was found to be high among chronic dialysis patients.

**Table 4:** Incidence, risk factors, and in-hospital mortality from liver abscess in different study population.

Author	Country	Study population	Annual incidence	Risk factor for LA	In-hospital
			(n/100,000)		mortality (%)
Hansen et al.^6^	Demark	County - based	1.1	DM, alcoholism	6
		General population	/100,000 general population		
Mølle et al.^7^	Demark	Nationwide-based	23.3		Alcoholic cirrhosis: 38.5%
		Liver cirrhosis population	/100,000 Liver cirrhosis		Non - alcoholic cirrhosis: 62.5%
Kaplan et al.^8^	Canada	City - based	2.3	Older age, DM, caner	10
		General population	/100,000 general population	men, liver tansplantation,	
Meddings et al.^9^	United States	Nationwide - based	3.6		6
		General population	/100,000 General population		
Tsai et al.^10^	Taiwan	Nationwide - based	11.5∼17.5	DM, cancer,	10.9
		General population	/100,000 general population	renal disease, pneumonia	
Yang et al.^11^	Taiwan	Hospital - based	NA	DM	33.3
		ESRD population	(Chart review)		
This study	Taiwan	Nationwide - based	224.9	PD, Older age, DM,	10.14
		ESRD population	/100,000 ESRD population	biliary tract disease	
				alcoholism, PCKD	

DM: diabetes mellitus; PCKD: polycystic kidney disease.

Besides the well-known risk factors in general population, this study found PCKD and PD to be as significant risk factors for liver abscess among ESRD dialysis patients. One study reported that 83% of PCKD between the ages of 15 and 46 years have hepatic cysts [19], entities associated with significant risk for liver abscess formation [20], [21]. Patients with PCKD in this study had nearly twice the risk of liver abscess than those without it. Dialysis modality was found to be an important risk factor. The most frequent infective complication of PD is peritonitis. Because intra-abdominal infection like bowel leakage or peritonitis is a major cause of liver abscess formation, it is reasonable to speculate that patients on PD have a higher risk of liver abscess than those on HD. Further studies are needed at next step to evaluate the mechanism underlying the development of liver abscess in PD patients based on the result of this study.

The in-hospital mortality in ESRD in this study (10.14%) was similar to that in the general Taiwanese population (10.9%) reported by Tsai et al. [5]. Because liver abscess is mostly an acute illness, the determinant factors for short-term mortality are always the severity of the acute illness and immediate therapy. In Taiwan, medical resource is very popular and sound. The medical expensed for patients with ESRD are covered by the National Health Insurance program. Drainage of liver abscess can be performed in most hospitals in Taiwan, especially for patients found to be refractory to antibiotic treatment. Thus, the average short term mortality rate between patient with and without ESRD is similar. Yang et al. [11], performing a study in a referral hospital (National Taiwan University Hospital) for the most seriously ill patients in Taiwan, reported that the mortality rate for liver abscess among ESRD patients to be 33.3%, much higher than the result reported in the current study based on nationwide and representative data. Thus, it is reasonable to speculate that in-hospital mortality due to liver abscess among ESRD patients would probably be high if acute treatment is performed in referral hospitals reserved for seriously ill patients.

Although the in-hospital death from liver abscess is most often acute, long-term mortality in our study population was often related to their ESRD status and baseline comorbidities, mostly DM and CLD. This study found DM to be an independent predictor of long-term mortality from liver abscess in ESRD dialysis patients. ESRD dialysis patients with DM generally have multiple comorbidities and have a higher risk of death [22]–[27]. Taiwan is an endemic area of chronic hepatitis because of a high prevalence of virus hepatitis B and C infection. Only few previous studies showed that the CLD to be an independent predictor of mortality. Liu et al. used United States Renal Data System to demonstrate liver disease is independent predictors of mortality (Relative risk 1.21, 95%CI: 1.15–1.27) [28]. Chien et al. further used Taiwan National Health Insurance Database [16] to report a high prevalence rate of liver cirrhosis among ESRD patients, and showed that liver cirrhosis is an important predictor of mortality in dialysis patients (HR 1.472, 95% CI: 1.329–1.634). ESRD patients with PCKD is a different disease entity from those without PCKD, who also frequently have DM, hypertension, glomerulonephritis and cardiovascular disease. ESRD patients with PCKD on dialysis have a significantly longer survival rate than those without PCKD [29]–[31]. We further analyzed the differences between ESRD patients with and without PCKD in our database. We also found that patients with PCKD tended to have less comorbidities than those without it. Only 13.2%, 16.5%, and 13.2% of patients with PCKD had DM, coronary artery disease, and heart failure, respectively. However, 49.7%, 23.5%, and 25.7% of patients without PCKD had DM, coronary artery disease, and heart failure, respectively. Perrone et al. [28], using data from the United States Renal Data System, found a lower mortality rate in PCKD dialysis patients than in nondiabetic dialysis patients. Zeier et al. [29] presumed that such an improvement may be a result of better hemoglobin levels on cardiac function.

This study has several limitations. First, similar to other studies using administrative data, our study has the problem of unmeasured confounders. Second, the comorbidities were identified based on claims data and ICD-9-CM diagnosis codes, some of which might have been incorrect. Third, our study lacked specific data on nutritional status, microbiology and biochemical data, as well as socioeconomic characteristics. The lack of microbiological data limited the interpretation of the impact of these findings in the settings. Finally, it is better to obtain more information when comparing ESRD patients with liver abscess to patients with liver abscess but no ESRD. However, our database (NHRI-NHIRD-99182) only included ESRD patients receiving dialysis, but no non-ESRD subjects for comparison. Thus, we were unable to use the current database to compare ESRD patients with liver abscess to patients with liver abscess but without ESRD.

In conclusion, there is a high incidence of liver abscess in ESRD dialysis patients, especially patients ≥ 65 years, receiving PD, and those with a history of DM, PCKD, malignancy, CLD, biliary tract disease, or alcoholism. More attention should be paid to awareness of liver abscess when treating these high-risk patients.
